# Second contralateral hip fractures reduce survival, mobility and daily activity : a matched pair analysis

**DOI:** 10.1007/s00402-026-06331-2

**Published:** 2026-05-07

**Authors:** Alisa Blattner, Florian Sabath, Timon Röttinger, Leonhard Lisitano, Edgar Mayr, Annabel Fenwick

**Affiliations:** Department of Trauma, Orthopedic, Plastic and Hand Surgery, University Hospital Augsburg, Stenglinstrasse 2, 86156 Augsburg, Germany

**Keywords:** Hip fractures, Frailty, Second contralateral hip fracture, Osteoporosis, Mortality

## Abstract

**Background:**

Hip fractures in older adults are associated with substantial morbidity, functional decline, and mortality. Patients who experience a first fragility fracture are at high risk of subsequent fractures, with repeated events further exacerbating functional impairment and survival outcomes. Second contralateral hip fractures, while clinically important, remain under-characterized in terms of timing, long-term functional impact, and mortality.

**Objectives:**

To investigate the incidence, timing, survival, mobility, and daily activity outcomes of second contralateral hip fractures using a matched pair analysis, and to situate these findings within the broader context of repeated fragility fractures.

**Methods:**

A retrospective cohort study was conducted at a single Level I trauma center, including all patients treated operatively for hip fractures (AO 31A1–A3, 31B) between January 2016 and June 2020. Patients with a second contralateral hip fracture were matched 1:1 with patients with a single fracture based on age, sex, fracture type, and Charlson Comorbidity Index. Demographic data, functional status (Barthel Index, Parker Mobility Score), walking aid use, living situation, and mortality were assessed at admission, discharge, and follow-up (minimum 3 years, maximum 7 years). Statistical analysis included paired tests and survival analysis.

**Results:**

Of 1,933 patients, 148 (7.6%) sustained a second contralateral hip fracture. Mean time to second fracture was 2.2 years, with 40% occurring within the first postoperative year. Time to death after the second fracture was significantly shorter (1.06 vs. 1.95 years, *p* < 0.001). Whilst one year mortality rate was significantly lower for the cohort of patients with a single hip fracture (18% vs. 33%, *p* < 0.03), the significance disappeared the longer the follow up time (2- year mortality: 37.9% vs. 48%; *p* < 0.098; 3- year mortality: 51.6% vs. 52.2%, *p* < 0.474).

Patients with second fractures demonstrated significantly lower Barthel Index (66.7 vs. 77, p=0.019) and Parker Mobility Score (5.39 vs. 6.84, p=0.06) at follow-up, indicating reduced independence and mobility. Discharge to rehabilitation after the first fracture was associated with higher risk of subsequent fracture (58.1% vs. 49.3%, p=0.035).

**Conclusion:**

Second contralateral hip fractures are underrecognized events that significantly reduce long-term mobility, independence, and survival time post-fracture. Most occur within the first two years after the initial fracture, highlighting a critical window for targeted monitoring, fall prevention, and optimized osteoporosis management. Early identification of high-risk patients, especially those discharged to rehabilitation, is essential to mitigate functional decline and mortality.

## Introduction

Hip fractures represent a critical event in older adults, often triggering functional decline, increased dependence, and high mortality [1, 2]. They are associated not only with acute surgical risks but also with long-term consequences, including loss of mobility, reduced independence in activities of daily living, and heightened risk for institutionalization [8]. While much research focuses on first hip fractures, a significant subset of patients—approximately 5–12%—experience a second contralateral hip fracture within subsequent years [3, 4]. With the global incidence of hip fractures rising due to aging populations [5, 6], the clinical and economic burden of second fractures is expected to grow substantially.

Second hip fractures are particularly concerning because they are often associated with worsened functional outcomes and increased healthcare costs comparable to first fractures [7, 8]. Previous studies have identified risk factors such as advanced age, female sex, delirium, frailty, and osteoporosis [3, 9, 10]. Despite improvements in osteoporosis diagnosis and treatment, treatment gaps remain substantial; only a small proportion of patients receive bone density testing or anti-osteoporotic therapy after an initial fracture [11–13]. Furthermore, interventions such as fracture liaison services, while effective, require investment and time to impact outcomes meaningfully.

Although multiple studies have investigated first hip fractures extensively, few have systematically examined second contralateral hip fractures. Existing literature often reports descriptive statistics or risk factors, but robust data quantifying the timing of second fractures, their impact on survival, and long-term functional outcomes are limited. Understanding these parameters is crucial for guiding follow-up care, secondary prevention strategies, and rehabilitation protocols.

To address this gap, we performed a matched pair analysis of patients with second contralateral hip fractures compared to matched controls with a single fracture. We aimed to quantify the timing of second fractures, assess their impact on mortality, mobility, and daily activity, and provide actionable insights for clinical management and prevention.

## Methods

### Study design and population

We conducted a retrospective cohort study at a Level I trauma center, including all operatively treated hip fracture patients (AO:31A1–A3, AO:31B) from January 2016 to June 2020. Exclusion criteria were conservative treatment, periprosthetic fractures, and polytrauma requiring surgery. Ethical approval was obtained (Ethics Committee 20–2155-101) in accordance with the Declaration of Helsinki.

### Data collection

Demographics, fracture type, comorbidities (Charlson Comorbidity Index, CCI [14]), surgical details, ambulatory status, Barthel Index [15, 16], Parker Mobility Score [17], walking aid use (categories: no aid, cane or crutches, walker, wheelchair, only transferor immobility), living situation (at home, assisted living of care home facility), and level of care (level 0: no care taking level, level 1: minor impairment of independence, level 2: moderate impairment of independence, level 3: severe impairment of independence, level 4: most severe impairment of independence, level 5: most severe impairment of independence with special care requirements) were collected at admission, discharge, and during follow-up. Follow-up occurred in 2023 via telephone with patients, relatives, or care facilities, collecting mobility, daily living activities, further falls, second fractures, and mortality (minimum 3-year follow-up, maximum 7 years).

Matched Pair Analysis.

Patients sustaining a second contralateral hip fracture formed the study group. Controls were matched 1:1 based on age, gender, fracture type, and CCI.

### Statistical analysis

Data were analyzed using IBM SPSS version 27. Normality was assessed, and comparisons used Student’s t-test, chi-square, Wilcoxon Rank Test, and Fisher’s exact test as appropriate. Significance was set at *p* < 0.05.

## Results

### Cohort characteristics

Of 1,933 eligible patients, 148 (7.6%) sustained a second contralateral hip fracture. Matched controls were identified for each patient (total *n* = 296). Most patients were female (74.3%), mean age 81.3 years (range 60–100). Fracture distribution: 63 femoral neck (42.6%), 74 pertrochanteric (50%), 11 subtrochanteric (7.4%). Mean CCI was 2.1 (range 0–7).

### Timing of second fractures

Exact dates were available for 126 patients (85%). The mean interval to the second fracture was 2.2 years (range 0–8). 40.2% occurred within one year, 20.6% in the second year, and nearly two-thirds within two years. Over the following years the number of second fractures continues to drop (year 3: 13.5%, year 4: 11.1%, year 5: 9.5%). Only five patients had fractures beyond five years.

### Mortality

**Fig. 1:**
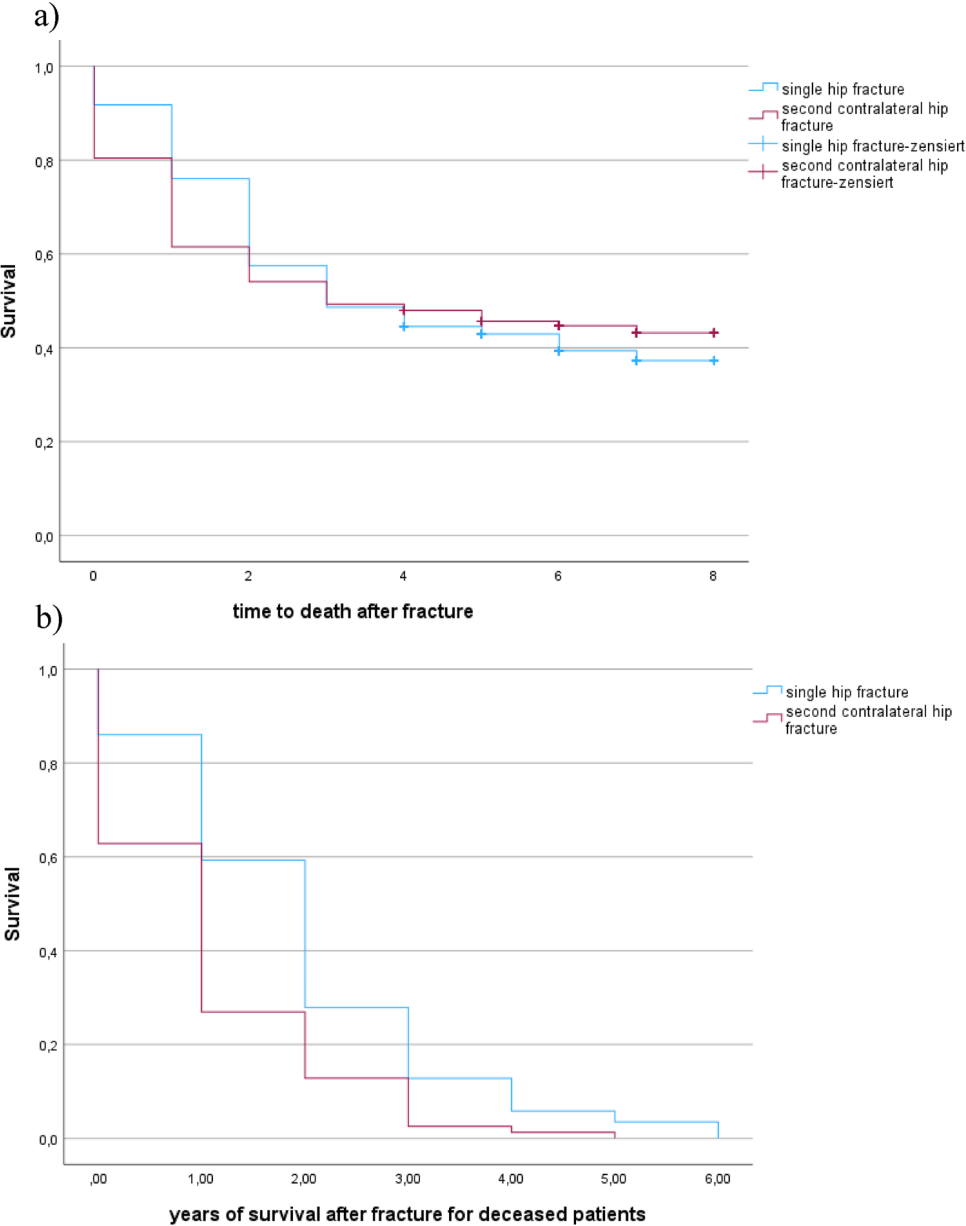
Survival rate comparison

Overall mortality did not differ significantly between groups (study: 55.4% vs. control: 60.1%, *p* = 0.24). However, time to death after the second fracture was significantly shorter (1.06 years vs. 1.95 years, *p* < 0.001), indicating accelerated decline post-second fracture, s. Figure 1. Comparable mortality rates were recorded at 1, 2 and 3 years. Whilst the mortality rate was significantly lower for patients with a single hip fracture one year postoperatively (18% vs. 33%, Kaplan Meier survival *p* < 0.03), the 2 and 3 year mortality rates approached each other steadily diminishing the difference between the two groups (2 years: 37.9% vs. 48%; *p* < 0.098; 3 years: 51.6% vs. 52.2%, *p* < 0.474).

## Functional outcomes

**Fig. 2:**
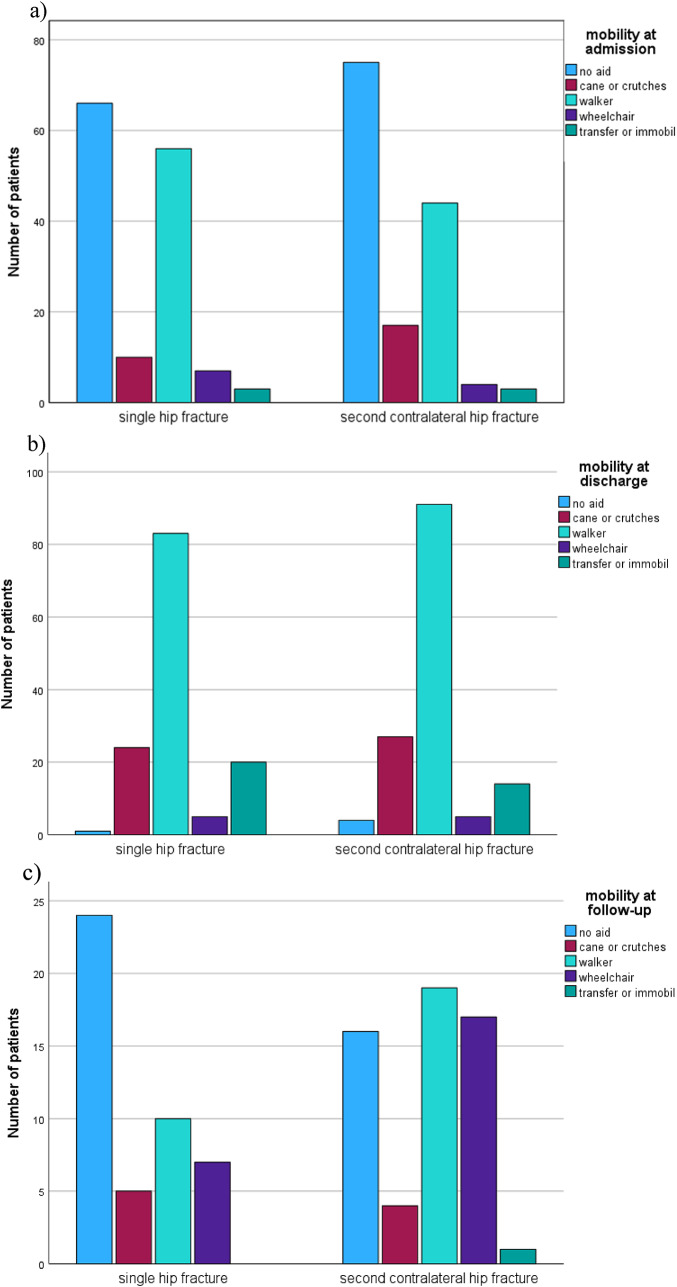
Level of mobility comparing patients with single and second contralateral hip fractures

**Fig. 3:**
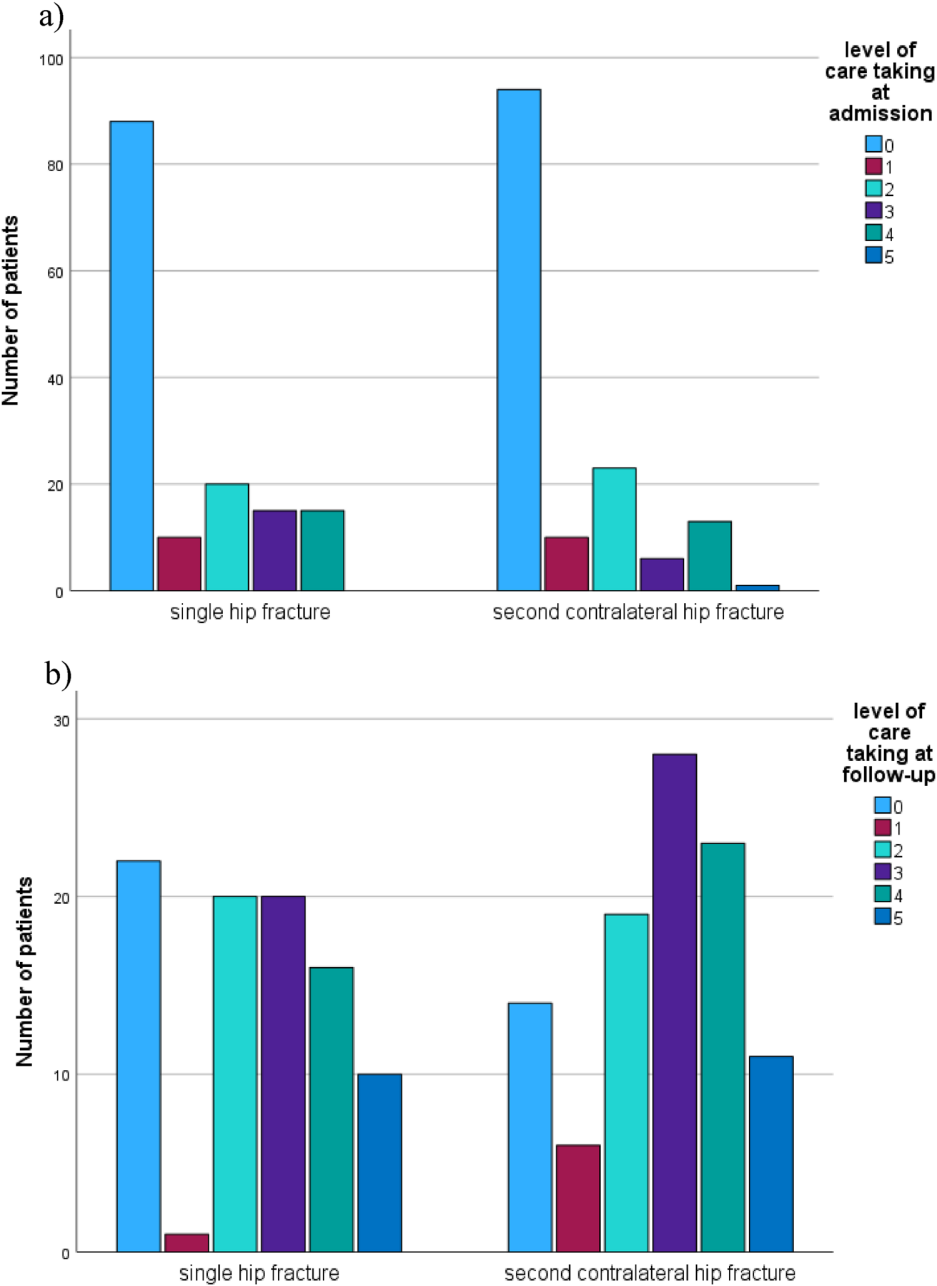
Level of care taking

**Fig. 4:**
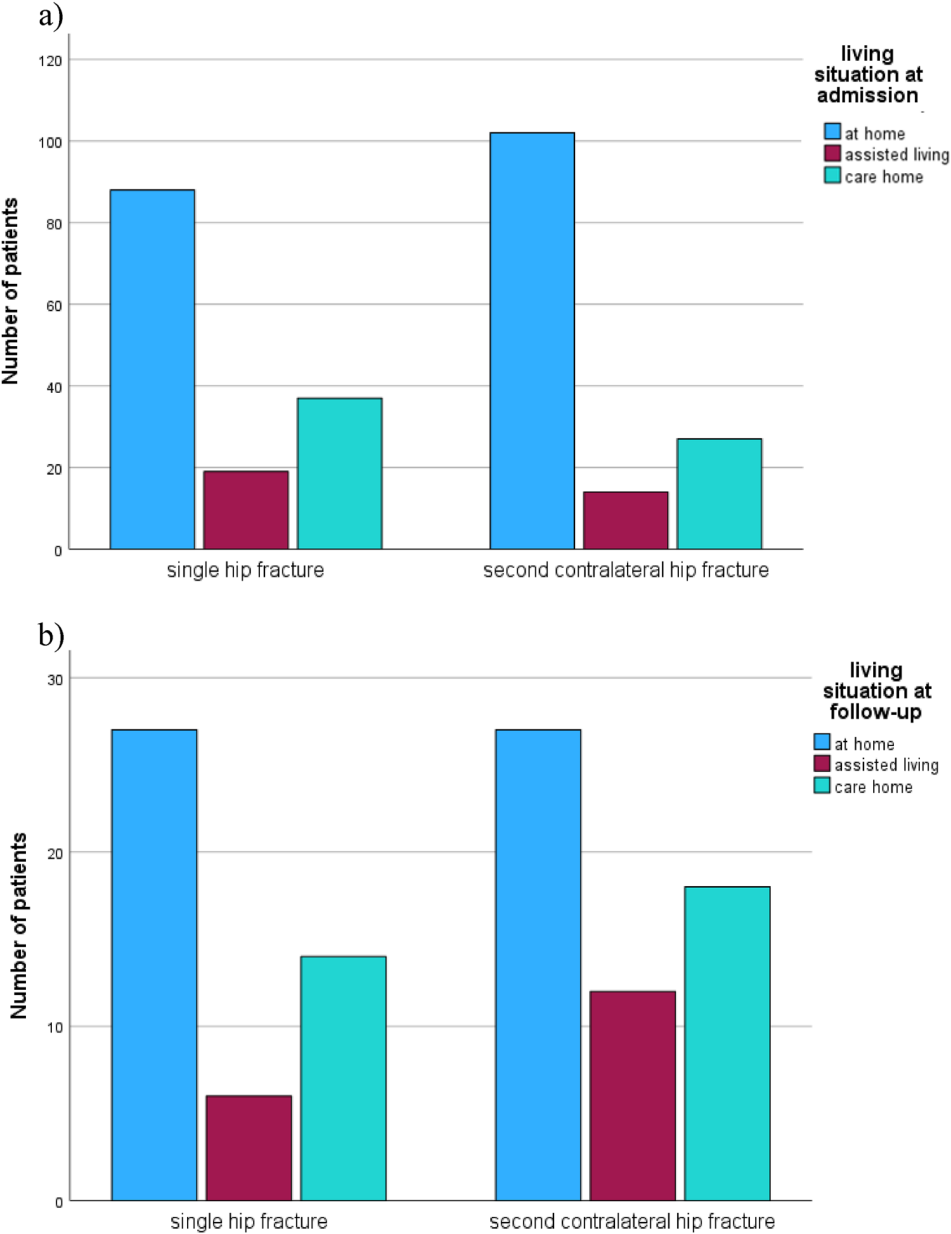
Comparison of the living situation of single hip fractures and contralateral second hip fractures At time of admission

**Fig. 5:**
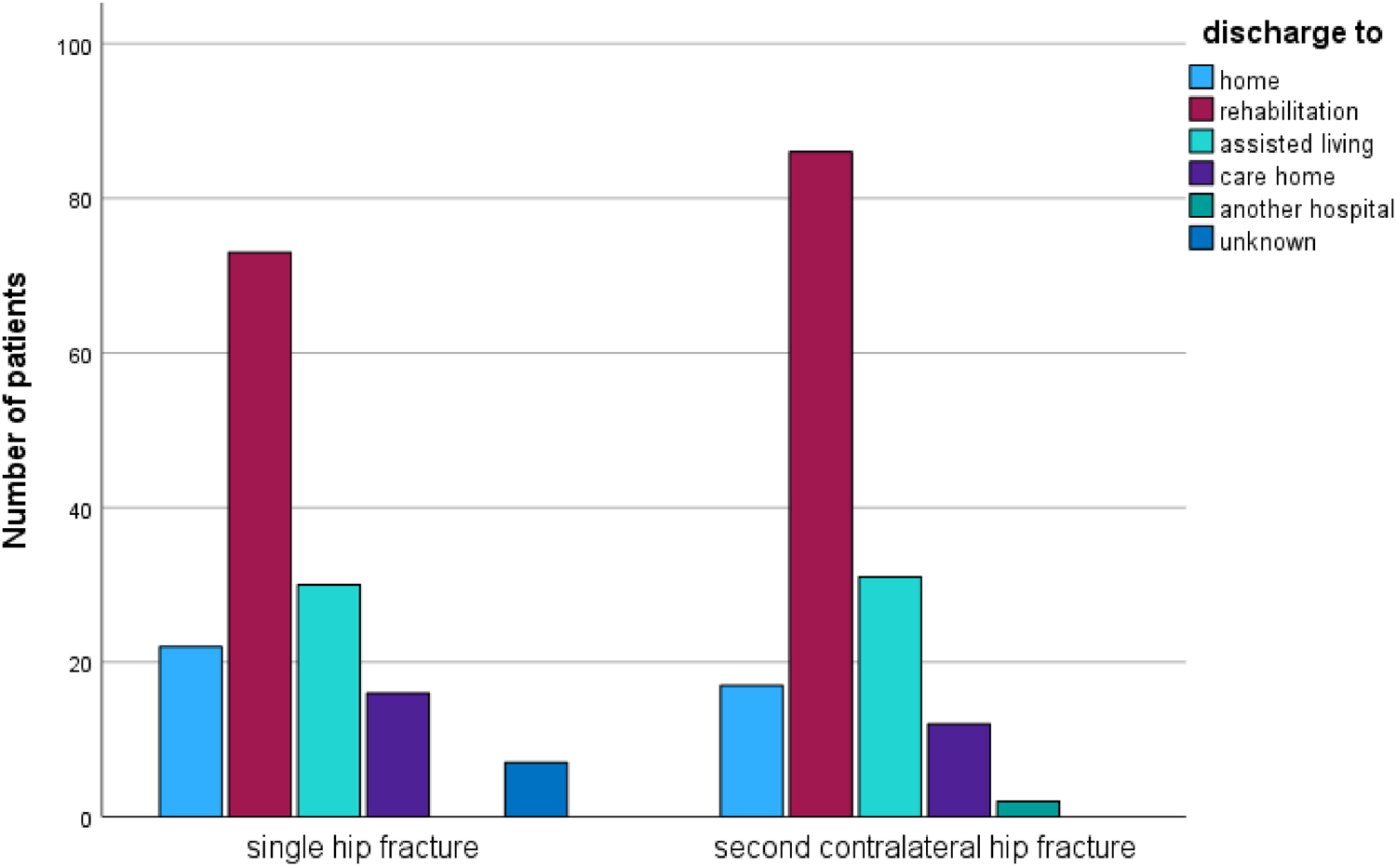
Distribution of destination of discharge within the compared groups single fracture and second contralateral fracture

Functional outcomes did not differ between the matched groups at discharge after the first fracture, as reflected by similar Barthel Index values (41.8 vs. 40.9, *p* = 0.79). However, at follow-up, patients who sustained only a single fracture demonstrated significantly greater independence, with higher Barthel Index scores compared to those with a second fracture (77 vs. 66.7, *p* = 0.019). Mobility, assessed using the Parker Mobility Score, was lower in patients with second fractures at telephone follow-up (5.39 vs. 6.84, *p* = 0.06), indicating reduced mobility and activity levels (Fig. 2). In addition, the use of walking aids and the level of care both increased following second fractures, further reflecting diminished independence (Figs. 2 and 3). Patients who were initially discharged to rehabilitation after the first fracture exhibited a higher risk of sustaining a second fracture compared to those discharged elsewhere (58.1% vs. 49.3%, *p* = 0.035). Finally, the living situation at follow-up also favored patients with a single fracture, suggesting better overall functional outcomes in this group (Figs. 4 and 5).

## Discussion

Hip fractures are widely recognized as a fundamental event in older adults, often heralding subsequent morbidity, functional decline, and increased mortality. While substantial literature has established that patients sustaining any fragility fracture are at elevated risk of additional fractures, our study specifically examines second contralateral hip fractures. Multiple prior investigations have shown that a history of more than one fragility fracture markedly increases the risk of subsequent fractures, accelerates functional decline, and elevates mortality [3, 7, 9, 18–21]. In this framework, our finding that 7.6% of our patients sustained a second contralateral hip fracture over a follow-up period of up to seven years aligns with the well-established concept that fragility fractures cluster and that a first fracture can predict imminent subsequent fractures.

Importantly, a substantial number of second hip fractures are potentially preventable. Pharmacological therapies for osteoporosis have been able to show a risk reduction of subsequent fragility fractures of up to approximately 50%, providing a strong evidence for early and consistent secondary prevention [22, 23]. Thus, the occurrence of a second hip fracture should not be viewed solely as an inevitable consequence of aging and frailty, but rather as, in many cases, a potentially modifiable outcome.

Our analysis demonstrates that the majority of second hip fractures occur within two years of the initial event, with a mean interval of 2.2 years, and approximately 40% occurring within the first postoperative year. This time scale coincides with prior studies across multiple fracture types, which have identified a high-risk period immediately following a first fragility fracture, often described as the “imminent risk” window [24–26]. By quantifying this pattern specifically for contralateral hip fractures and within a matched cohort, our study reinforces and contextualizes these observations. While the risk of a second fracture during this highlighted period is predictable, our findings highlight that second hip fractures continue to have profound consequences for survival and long-term functional independence.

We also observed that nearly half of patients sustained the same fracture type in the contralateral hip, whereas the remaining patients experienced a different fracture type. This pattern aligns with previous observations by Lee and Kwek [27], who reported a similar distribution in a Singaporean elderly cohort. Recognizing that the second fracture may mirror the anatomical pattern of the first has implications for risk stratification and fall-prevention strategies, as patients with an initial femoral neck or pertrochanteric fracture may be predisposed to similar biomechanical vulnerabilities in the contralateral hip.

By matching patients on age, sex, fracture type, and comorbidity burden, we were able to isolate the effects of a second fracture from confounding baseline characteristics. At long-term follow-up, patients with a second fracture exhibited lower Barthel Index scores and reduced Parker Mobility Scores, indicating significant declines in independence and mobility compared to matched controls. These functional decrements mirror patterns described in broader fragility fracture populations, in which repeated fractures accelerate functional decline and increase dependency [7, 18–21]. Notably, no significant differences were observed at discharge after the first fracture, suggesting that the second event itself contributes directly to the worsening outcomes.

The association between initial discharge to rehabilitation and increased risk of a second fracture aligns with prior observations that frailty and impaired baseline mobility predispose patients to subsequent fractures [9, 21]. Rather than implicating rehabilitation as causative, this finding highlights the need for targeted post-discharge monitoring, early fall-prevention strategies, and rigorous osteoporosis management. Despite advances in pharmacologic therapy for osteoporosis, secondary prevention remains underutilized, with only a minority of patients receiving anti-osteoporotic therapy prior to the second fracture [3, 28]. Importantly, all older adults following a hip fracture are considered at high risk for subsequent fragility fractures and should be evaluated for pharmacologic therapy unless contraindicated, in line with international guidelines.

In this study, we were unable to reliably compare outcomes between patients who did and did not receive pharmacological therapy after the first fracture due to limited and inconsistent data on treatment initiation, adherence, and duration. This represents an important limitation of our analysis. Future prospective studies with detailed medication data are needed to better quantify the real-world effectiveness of post-fracture pharmacological interventions specifically in preventing second hip fractures.

While our findings contribute detailed functional and survival data specifically for second contralateral hip fractures, they should be interpreted within the broader context of fragility fracture literature. Numerous studies have documented the cumulative burden of multiple fragility fractures across various skeletal sites, consistently showing heightened fracture risk, reduced mobility, and increased mortality [24, 25, 29, 30]. In the Dubbo Osteoporosis Epidemiology Study, a history of prior fragility fracture more than doubled the risk of future fractures and was associated with excess mortality across bone density strata [31]. Large registry studies have also shown markedly increased fracture risk shortly after an index fragility fracture, with subsequent fractures contributing to higher mortality risk extending beyond the first fracture period [32, 33]. Our study complements these findings by providing a focused, long-term, matched pair analysis of the specific trajectory of patients with a second contralateral hip fracture, rather than evaluating fragility fractures in aggregate.

This study has limitations. Its retrospective, single-center design may limit generalizability, and detailed data on osteoporosis diagnostics, pharmacologic treatment adherence, and biochemical parameters were not consistently available. Some functional assessments relied on telephone follow-up, which could introduce reporting bias. Despite these limitations, the extended follow-up, rigorous matching, and inclusion of functional outcome measures provide robust, clinically meaningful insights.

### Clinical implications

Our results emphasize the need for early identification and management of all patients at risk for subsequent fractures, particularly during the first two years after an initial hip fracture, which represents a critical period of heightened vulnerability. Clinicians should prioritize comprehensive osteoporosis assessment and timely initiation of pharmacologic therapy for all older adults following a hip fracture, as they are universally considered high risk for future fragility fractures, unless contraindicated. Structured fall-prevention strategies and individualized rehabilitation plans remain essential, especially for patients discharged to post-acute care facilities, to preserve mobility and independence.

## Conclusion

Second contralateral hip fractures represent a clinically significant and underrecognized event within repeated fragility fractures. While the increased risk of subsequent fractures shortly after an initial event is well-established, our matched cohort analysis demonstrates that these fractures are associated with accelerated functional decline and reduced survival. Most second fractures occur within the first two years after the initial hip fracture, defining a critical period for intensified monitoring, fall prevention, and optimization of osteoporosis management. Early identification of high-risk patients, prompt post-fracture interventions, and evaluation for pharmacologic therapy in all older adults are essential to mitigate the cumulative impact of repeated fractures on mobility, independence, and survival.

## Data Availability

The datasets used and/or analysed during the current study available from the corresponding author on reasonable request.
